# Risk Factors Associated with Recurrent Diarrheal Illnesses among Children in Kabul, Afghanistan: A Prospective Cohort Study

**DOI:** 10.1371/journal.pone.0116342

**Published:** 2015-02-13

**Authors:** Adam R. Aluisio, Zabihullah Maroof, Daniel Chandramohan, Jane Bruce, Mohammad I. Masher, Semira Manaseki-Holland, Jeroen H. J. Ensink

**Affiliations:** 1 Department of Disease Control, London School of Hygiene and Tropical Medicine, London, United Kingdom; 2 Department of Emergency Medicine, Division of International Emergency Medicine, SUNY Downstate Medical Center, Brooklyn, New York, United States of America; 3 Department of Paediatrics, Kabul Medical University, Kabul, Afghanistan Department of Pediatric Endocrinology, Royal Manchester Children's Hospital, Manchester, United Kingdom; 4 School of Health and Population Sciences, College of Medical and Dental Sciences, University of Birmingham, Birmingham, United Kingdom; Université Paris Descartes; AP-HP, Groupe Hospitalier Cochin-Saint-Vincent-de-Paul, FRANCE

## Abstract

**Introduction:**

Childhood diarrheal illnesses are a major public health problem. In low-income settings data on disease burden and factors associated with diarrheal illnesses are poorly defined, precluding effective prevention programs. This study explores factors associated with recurrent diarrheal illnesses among children in Kabul, Afghanistan.

**Methods:**

A cohort of 1–11 month old infants was followed for 18 months from 2007–2009. Data on diarrheal episodes were gathered through active and passive surveillance. Information on child health, socioeconomics, water and sanitation, and hygiene behaviors was collected. Factors associated with recurrent diarrheal illnesses were analyzed using random effects recurrent events regression models.

**Results:**

3,045 children were enrolled and 2,511 (82%) completed 18-month follow-up. There were 14,998 episodes of diarrheal disease over 4,200 child-years (3.51 episodes/child-year, 95%CI 3.40–3.62). Risk of diarrheal illness during the winter season was 63% lower than the summer season (HR = 0.37, 95%CI 0.35–0.39, P<0.001). Soap for hand washing was available in 72% of households and 11.9% had toilets with septic/canalization. Half of all mothers reported using soap for hand washing. In multivariate analysis diarrheal illness was lower among children born to mothers with post-primary education (aHR = 0.79, 95%CI 0.69–0.91, p = 0.001), from households where maternal hand washing with soap was reported (aHR = 0.83, 95%CI 0.74–0.92, p<0.001) and with improved sanitation facilities (aHR = 0.76, 95%CI 0.63–0.93, p = 0.006). Malnourished children from impoverished households had significantly increased risks for recurrent disease [(aHR = 1.15, 95%CI 1.03–1.29, p = 0.016) and (aHR = 1.20, 95%CI 1.05–1.37, p = 0.006) respectively].

**Conclusions:**

Maternal hand washing and improved sanitation facilities were protective, and represent important prevention points among public health endeavors. The discrepancy between soap availability and utilization suggests barriers to access and knowledge, and programs simultaneously addressing these aspects would likely be beneficial. Enhanced maternal education and economic status were protective in this population and these findings support multi-sector interventions to combat illness.

**Trial Registration:**

www.ClinicalTrials.gov NCT00548379 https://www.clinicaltrials.gov/ct2/show/NCT00548379

## Introduction

Diarrheal diseases account for over 700,000 child deaths annually, with 98% of these occurring in low- and middle-income countries (LMIC).[1] In 2010, 1.7 billion cases of diarrhea were estimated to occur in children.[2] Although the mortality and disease incidence have declined, the public health burden in children remains substantial; resulting in malnutrition, impaired development and reduced vaccine efficacy.[3–6]

Infectious diarrhea is caused by a variety of pathogens. In children most cases of moderate-to-severe diarrhoea are attributable to four pathogens: rotavirus, *Cryptosporidium*, enterotoxigenic *E*. *coli*, and *Shigella*.[7] Diarrhoegenic agents are transmitted through fecal-oral routes, which include: transmission by flies, ingestion of contaminated food, or water, and person-to-person contact.

World Health Organization (WHO) recommendations for prevention of childhood diarrhea promote vaccinations, child nutrition, and interruption of fecal-oral transmission routes. Prevention strategies for interrupting fecal-oral transmission routes focus on hand washing, sanitation, and access to sufficient safe water. Although improved hygiene, water supply and sanitation could prevent 95% of all diarrhea cases,[8] and the fact that safe and water and sanitation are acknowledged as a basic human right,[9] over a 750 million people still lack access to improved water supply, and 2.5 billion people lack access to improved sanitation[10].

Globally, Afghanistan has the fourth highest diarrheal mortality rates and approximately nine percent of all deaths among children 1–59 months of age are due to diarrheal diseases.[11,12] However, with the exception of a small number of studies,[13–15] there exists limited data pertaining to diarrheal illnesses in Afghan children. With multiple transmission routes and etiologic pathogens associated with childhood diarrhea it is key to identify specific factors associated with disease in order to identify setting appropriate effective interventions. This becomes even more important, when financial resources are limited and when depleted post-conflict infrastructure exists, as is the case in Afghanistan, and Kabul in particular.[16] This study aimed to describe the incidence of recurrent diarrhea and identify factors associated with enteric illnesses among children in Kabul, with the goal of informing preventative measures in the region.

## Methods

### Ethics and reporting

Research approval was obtained from the Ethics and Review Board of the Ministry of Public Health of Afghanistan (Reference: 422328) and the Ethics Committee of the London School of Hygiene and Tropical Medicine (Application no. 5117). Written informed consent was obtained from the mother and father, or other head of household for all enrolled children where the individual was not adequately literate, the details of the consent were read and explained to the consenting caregiver by study staff and all questions were answered. Reporting guidelines for observational studies were followed.[17]

### Data collection

Data were collected between November 2007 and June 2009 in five districts from within Kabul, Afghanistan. The districts comprise the central city and adjacent embankment and represent Kabul’s socio-economically deprived inner-city population. All of the districts were within the catchment area of the study hospital. The data was derived from a randomized controlled trial designed to evaluate the impact of supplementation with vitamin D on the incidence of childhood pneumonia and diarrheal illnesses.[18,19] Details on the trial design, household selection, inclusion and exclusion criteria have been presented previously.[18]

A total of 3,045 infants aged one to eleven months and residing in households located within the study districts were enrolled, and followed for 18 months prospectively. An episode of diarrheal illness was defined as a child having three or more loose stools in a 24 hour period.[11] A symptom free period of ≥72 hours was required to define a unique recurrent event otherwise the illness was considered part of the prior diarrheal episode.[20] Data on the occurrence of, and risk factors associated with diarrhea were collected through fortnightly household visits. During visits, children underwent a physical examination, and their health history was gathered by caregiver report. Recall of defecation history was based on the 24-hour period preceding each visit. Passive surveillance of self-referring children was undertaken at the Maiwind Teaching Hospital, the primary health center serving the study districts. During each clinic evaluation trained pediatricians completed standardized data forms assessing the number of loose/liquid stools during the 24-hours prior to presentation. If a study child was absent during a home visit, an absentee form was completed, and information regarding child health and treatment during the period was gathered when the child was contacted in the subsequent visit.

At enrollment, data on household socio-demographic characteristics and infant health were collected. Additional cross-sectional data was gathered during follow-up. Child feeding modality was categorized based on maternal report over one week prior to sampling as exclusive breastfeeding, mixed breastfeeding, or replacement feeding. Nutritional status was assessed using weight-for-age z-scores (WAZ) calculated at four time points during follow-up. Water and sanitation characteristics included information on hand washing practices, household sanitation facilities, food storage and water access and treatment. Impoverished households were defined based on World Bank standards (daily household income per family member utilized).[21]

### Data analysis

Data analysis was performed using STATA version 10.0 (College Station, USA). In estimating child-time at risk, participants were censored four days after each episode to account for mean illness duration.[20] Diarrheal episodes with repeat visits were excluded. Children absent from surveillance for greater than 45 days were censored at the time of their last recorded contact, and if subsequently relocated were reentered into follow-up at that time.

To assess for changes in the incidence of diarrheal illness that occur with development, child age groups were stratified as: ≤ 6 months, > 6 months to ≤ 12 months and > 12 months.[22] Children found to have WAZ ≤ -2 during follow-up were classified as malnourished in analyses.[23] To explore the role of climate variation on diarrheal disease a seasonal variable was derived based on months of follow-up.[24]

Distribution of the characteristics of the overall cohort and the subset of children who were lost-to-follow-up were compared. Significant differences between children who completed follow-up and those lost-to-follow-up were assessed using Pearson X^2^ and independent sample t-tests. The cohort was analyzed using recurrent events Poisson regression modeling. A random effects model was used to account for intra-child clustering of events (participant heterogeneity).[25] Incidence rates of diarrheal illnesses with corresponding 95% confidence intervals (95% CI) were calculated using linear combinations of coefficients.

Variables were explored in univariate recurrent events analyses to calculate hazard ratios (HR) with 95%CI. Multivariate forward stepwise Poisson regression models yielding adjusted hazard rations (aHR) were built utilizing all assessed factors and evaluated through likelihood ratio testing (LRT) with a p value <0.05 considered significant for inclusion. Child age and season were defined *a priori* to be included in all analyses. Child age was significantly associated with feeding modality, and age categorization was used in multivariate models. Similarly, maternal reports of hand washing with soap before eating, and after toilet use, where highly associated and only post toilet use was used in the recurrent events analyses. Goodness-of-fit of the final multivariate model was assessed through LRT and was found to be robust (p <0.05). The randomization variable was added to the final model to assess for effect on outcomes and no alterations to estimates were found.

## Results

**Fig 1:**
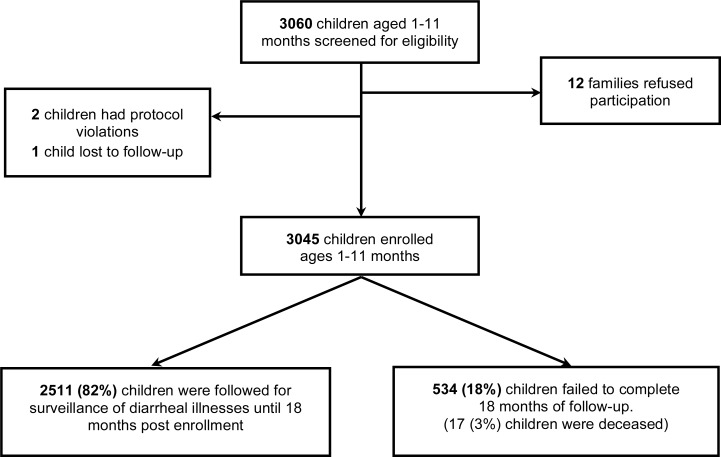
Study population.

Among the 3,060 infants screened 3,045 were enrolled with 2,511 (82%) followed for 18 months. There were 534 (18%) children lost to follow-up, of which 17 (3%) died (Fig. 1). Ten deaths were attributed to pneumonia/septicemia and seven were due to congenital or accidental causes. There were 14,998 episodes of diarrheal illnesses diagnosed; 6,094 (40.6%) episodes from home visit data, and 8,904 (59.4%) from hospital assessments. Seventy-eight (0.5%) children required inpatient treatment for a diarrheal episode. Cumulative follow-up time was 4200.0 child-years. The observed incidence of diarrheal illness was 3.51 episodes per child-year (95%CI 3.40–3.62).

Mean age at enrollment was 6.2 (SD±3.1) months. The majority of children ≤ 6 months were either exclusive breast fed, or received a mixed mode of feeding. Approximately 16% of the children were malnourished, and half of children had received their first measles vaccination at time of enrollment. The majority (81.2%) of mothers had none or only primary education. No significant differences were found between children completing the study and those that were lost-to-follow-up, with the exception of a greater proportion of children completing the study had received their measles vaccination at enrollment (p<0.001).

Approximately half of households obtained water from a piped source and the majority (72.7%) reported no drinking water treatment. Most families (88.1%) reported us of an open/ground toilet (defecation at designated places outside of the home). Although soap was present in over 70% of all household, only 38.8% of mothers reported hand washing with soap after using the toilet (Table 1).

**Table 1:** Characteristics of the study population.

Characteristics	Overall Cohort (N = 3045)	Lost-to-follow-up (n = 534)
	n(%)/mean(±SD)	n(%)/mean(±SD)
*Child*		
Gender		
female	1455(47.8)	239(44.8)
male	1590(52.2)	295(55.2)
Age at enrollment (months)	6.2(±3.1)	6.3(±3.2)
Feeding modality at ≤ 6 months of age		
exclusive breast feeding	432(43.3%)	69(43.3%)
mixed breast feeding	533(53.4%)	73(49.2%)
non-breast fed	33(3.3%)	6(4.1%)
Weight-for-age z-scores		
> -2	2325(78.4%)	456(94.0%)
≤ -2	484(16.3%)	32(6.0%)
Sleeps		
alone	2227(88.1)	345(89.4)
with ≥ 1 other child	302(11.9)	41(10.6)
Received first measles vaccination		
no	1570(51.6)	396(74.2)
yes	1475(48.4)	138(25.8)
*Maternal*		
Age		
≤ 15 years	209(6.9)	44(8.3)
> 15 years	2833(93.1)	489(91.7)
Highest education		
primary or none	2473(81.2)	424(79.4)
Secondary or greater	572(18.8)	110(20.6)
*Paternal*		
Highest education		
none	888(29.2)	175(32.8)
primary or greater	2156(70.8)	358(67.2)
Current employment		
no	177(5.8)	42(7.9)
Yes	2868(94.2)	492(92.1)
Impoverished households^a^		
no	364(20.6)	16(25.4)
Yes	1406(79.4)	47(74.6)
*Water and sanitation*		
Water source type		
Piped	1231(47.5)	91(36.6)
open well	742(38.6)	79(31.7)
tube well	618(23.9)	79(31.7)
Drinking water treatment		
None	1974(72.7)	184(71.0)
Chlorine	305(11.2)	32(12.4)
Boiling	436(16.1)	43(16.6)
Water source distance		
piped to home	691(27.1)	29(22.1)
well in home	1003(39.3)	81(61.8)
well outside of home	857(33.6)	21(16.1)
Food storage		
refrigerator	264(10.7)	12(5.2)
cold water	579(23.4)	67(29.0)
Nothing	1627(65.9)	152(65.8)
Toilet type		
open toilet/ground	2380(88.1)	530(88.6)
toilet (septic/canalization)	323(11.9)	68(11.4)
Mother reports washing hands with soap prior to eating		
No	1136(51.6)	100(42.2)
Yes	1065(48.4)	137(57.8)
Mother reports washing hands with soap post-toilet		
No	1289(61.2)	136(60.2)
Yes	817(38.8)	90(39.8)
Number rooms in home		
< 5	2654(89.9)	423(91.6)
≥ 5	299(10.1)	39(8.4)

*a*. Impoverished household defined as households that reported living on less than 1.25 US dollars per person per day.[21]

### Diarrheal Risk factors

**Fig 2:**
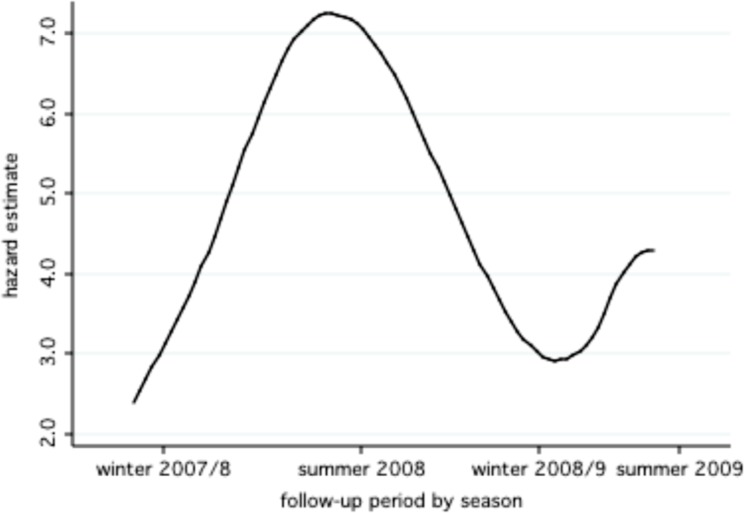
Estimated hazards for diarrheal illness by season^a^. a. Hazards estimates are adjusted for child age.

The highest risk of diarrheal illnesses were during the summer months (incidence 5.71 episodes per child-year (95%CI 5.48–5.96) and lowest during winter months (2.12 episodes per child-year (95%CI 2.02–2.21). The seasonal trends in hazards are illustrated in Fig. 2. The risk of diarrhea was 63% lower in winter as compared to summer (HR = 0.37, 95%CI 0.35–0.39, p<0.001).

Among children less than six months of age the incidence of diarrheal episodes was 2.35 per child-year (95%CI 2.15–2.57). For children aged six months to less than one year, the incidence was 3.89 episodes per child-year (95%CI 3.73–4.06). In univariate analysis, the risk of diarrheal illness was 60% greater among children aged six months to one year in comparison to those less than six months of age. For children greater than one year of age, the incidence of diarrheal illness was 3.48 episodes per child-year (95%CI 3.36–3.60) with a 48% greater risk of recurrent episodes. Malnourished children and those from impoverished households had a greater risk for recurrent illnesses. Children who were born to mothers with greater than primary education had a lower risk recurrent events as compared to those born to mothers with primary education or less.

Among children from homes using wells the risk of diarrheal illnesses was lower in comparison to a piped water source. Water treatment with chlorine was found to confer a reduced risk of diarrheal illnesses however no significant difference was found when treatment with boiling was reported. Food storage with refrigeration, having an in-home well, use of a toilet with septic/canalization and maternal hand washing with soap post-toilet use were all associated with a reduced risk of diarrheal disease (Table 2).

**Table 2:** Univariate analysis for risk of diarrheal illness.

Factor	Events	Child-years	Incidence per child-year (95% CI)	HR (95% CI)	P value
*Child*					
Gender					
Female	6843	2000.0	3.16(2.85–3.50)	1.00	
Male	8053	2200.0	3.38(3.23–3.55)	1.07(1.01–1.14)	0.034
Age (months)					
< 6	563	224.8	2.35(2.15–2.57)	1.00	
> 6 to < 12	3913	992.6	3.89(3.73–4.06)	1.65(1.51–1.81)	<0.001
> 12	10420	3000.0	3.48(3.36–3.60)	1.48(1.35–1.62)	<0.001
Weight-for-age z-scores					
> -2	10753	3200.0	3.37(3.26–3.49)	1.00	
< -2	3861	959.1	3.98(3.69–4.30)	1.19(1.10–1.29)	<0.001
Sleeps					
Alone	11027	3100.0	3.48(3.35–3.61)	1.00	
with >1 other child	1684	424.1	3.97(3.60–4.38)	1.14(1.03–1.27)	0.014
> 1 measles vaccination					
no	7226	2000.0	3.49(3.34–3.65)	1.00	
Yes	7670	2200.0	3.53(3.38–3.69)	1.01(0.95–1.08)	0.76
*Maternal*					
Age (years)					
<15 years	1144	287.6	4.32(3.39–5.49)	1.00	
>15 years	13743	3900.0	3.88(3.44–4.37)	0.90(0.80–1.02)	0.09
Highest education					
primary or none	12909	3400.0	3.74(3.61–3.87)	1.00	
post primary	1987	782.0	2.51(2.33–2.71)	0.67(0.62–0.73)	<0.001
*Paternal*					
Employed					
no	948	241.0	3.86(3.40–4.40)	1.00	
Yes	13948	4000.0	3.48(3.38–3.61)	0.90(0.79–1.03)	0.13
Impoverished households^a^					
no	1555	542.9	2.86(2.61–3.13)	1.00	
Yes	7870	2100.0	3.74(3.58–3.91)	1.31(1.18–1.45)	<0.001
*Water and sanitation*					
Type of water source					
Piped	7334	1800.0	4.02(3.84–4.22)	1.00	
open well	3461	1100.0	3.17(2.98–3.38)	0.79(0.73–0.85)	<0.001
tube well	2955	899.0	3.27(3.06–3.51)	0.81(0.75–0.88)	<0.001
Drinking water treatment					
None	10732	2900.0	3.69(3.55–3.83)	1.00	
Chlorine	1286	442.6	2.91(2.63–3.22)	0.79(0.71–0.88)	<0.001
Boiling	2264	642.5	3.51(3.24–3.81)	0.95(0.87–1.04)	0.27
Distance to water source					
piped to home	3804	1000.0	3.67(3.44–3.91)	1.00	
well in home	4428	1500.0	2.98(2.82–3.15)	0.81(0.75–0.88)	<0.001
well outside of homed	5395	1300.0	4.20(3.97–4.44)	1.14(1.05–1.24)	0.002
Food storage					
Refrigerator	1174	393.1	2.99(2.68–3.33)	1.00	
cold water	3236	843.3	3.58(3.43–3.73)	1.19(1.06–1.34)	0.003
none used	8542	2400.0	3.83(3.57–4.12)	1.28(1.13–1.47)	<0.001
Toilet type					
open toilet	12948	3500.0	3.69(3.56–3.82)	1.00	
toilet (septic/canalization)	1291	470.2	2.75(2.49–3.03)	0.75(0.67–0.83)	<0.001
Mother reports washing hands with soap post-toilet use					
No	7360	1900.0	3.89(3.71–4.07)	1.00	
Yes	3983	1200.0	3.32(3.13–3.52)	0.85(0.79–0.92)	<0.001
Number rooms in home					
< 5	13435	3700.0	3.57(3.46–3.69)	1.00	
> 5	1283	430.4	2.97(2.68–3.30)	0.83(0.75–0.93)	0.001

*a*. Impoverished household defined as households that reported living on less than 1.25 US dollars per person per day.[21]

In recurrent event multivariate analysis malnourishment and being from an impoverished household were associated with an increased risk of childhood diarrheal illnesses at 15% and 20% respectively (aHR = 1.15, 95%CI 1.03–1.29, p = 0.016 and aHR = 1.20, 95%CI 1.05–1.37, p = 0.006). Level of maternal education, maternal hand washing with soap post-toilet, and use of a toilet with septic/canalization were found to be protective against recurrent illnesses. Maternal education post primary school was associated with 21% lower risk (aHR = 0.79, 95%CI 0.69–0.91, p = 0.001), and hand washing with soap a 17% reduction (aHR = 0.83, 95%CI 0.74–0.92, p<0.001). Children from households using toilets with septic/canalized systems were had a 24% lower risk of diarrheal illnesses (aHR = 0.76, 95%CI 0.63–0.93, p = 0.006).

A trend of reduced risk was found among households using an open well versus a piped water source (aHR = 0.87, 95%CI 0.76–1.00, p = 0.053). No significant association was found in relation to diarrheal illnesses and tube wells. Treatment of drinking water, food storage, distance to water source, sleeping with other children and number of rooms in the home were not significantly associated with risk of diarrheal illness in multivariate analysis (Table 3).

**Table 3:** Multivariate analysis for risk of diarrheal illness.

Factor	aHR	(95% CI)	p value
Weight-for-age z-scores			
> -2	1.00		
≤ -2	1.15	(1.03–1.29)	0.016
Sleeps			
alone	1.00		
with ≥1 other child	1.05	(0.90–1.22)	0.550
Maternal education			
primary or none	1.00		
post primary	0.79	(0.69–0.91)	0.001
Mother reports washing hands with soap post-toilet use			
no	1.00		
yes	0.83	(0.74–0.92)	<0.001
Type of water source			
piped	1.00		
open well	0.87	(0.76–1.00)	0.053
tube well	0.95	(0.79–1.14)	0.576
Distance to water source			
piped to home	1.00		
well in home	0.94	(0.80–1.12)	0.507
well outside of home	1.08	(0.95–1.23)	0.239
Drinking water treatment			
None	1.00		
chlorine	0.88	(0.73–1.06)	0.187
boiling	0.89	(0.77–1.03)	0.106
Toilet type			
open toilet/ground	1.00		
toilet (septic/canalized)	0.76	(0.63–0.93)	0.006
Food storage			
refrigerator	1.00		
cold water	0.92	(0.77–1.10)	0.359
nothing or fresh foods	0.99	(0.81–1.21)	0.946
Impoverished households^a^			
no	1.00		
yes	1.20	(1.05–1.37)	0.006

*a*. Impoverished household defined as households that reported living on less than 1.25 US dollars per person per day.[21]

## Discussion

This study provides new findings pertinent to prevention of childhood diarrheal disease in both the global and Afghanistan specific settings. In this cohort the incidence of diarrheal illness was identified in a population that has previously lacked contemporary statistics, and the seasonal profile showed that disease burden is greatest during the winter months. Water and sanitation risk factors in relation to hand washing, and use of improved sanitation facilities were of key importance in mitigating the risk of diarrheal illness in this population.

The overall incidence of disease for children between one and 29 months of age agrees with global childhood estimates.[22] The incidence in this cohort also coincides with research from geographically, and culturally similar regions. In a study from Pakistan an incidence of 3.5 episodes per child-year was found among children less than three years of age.[26] Additionally, a study from Egypt reported an incidence of 3.6 episodes per child-year.[27] In this cohort the lowest incidence of diarrheal illness was found among children less than six months of age and the highest was present among those aged between six months and one year. These trends reproduce age band specific variations in the incidence of diarrheal disease reported in secular analyses of pooled global estimates.[22,28,29] The similarity between the incidences found in this prospective cohort and other works suggests validity in the findings.

As in other settings, in the present analysis greater maternal education was found to have a protective effect against childhood illness.[13,30] Although these data do not allow for identification of the specific aspects of education accounting for the protective effect it is possible that similar to other low-income settings education cultivated more accurate health knowledge, receptivity to health messages and improved communication abilities in this cohort.[31] As enhancement of maternal education in coordinating effective interventions is a component of The integrated Global Action Plan for Pneumonia and Diarrhoea, our findings provide further evidence supporting the importance of maternal education in reducing child morbidity in high burden regions.[32]

In meta-analyses, interventions of hand washing with soap and use of improved sanitation facilities have each been associated with greater than 30% reductions in disease risk.[33,34] In this study, hand washing was associated with a 15% reduction and household use of a toilet with septic/canalization a 24% reduction. Although these reductions are lower than pooled estimates, their agreement with the reported protective trends and significance in multivariate analysis demonstrates their importance in the setting of Kabul.

In this population, maternal hand washing with soap was reported by less than half of mothers however fieldworkers observed soap availability in 72% of households. This discrepancy between availability and utilization indicates that lack of use may be a function of both barriers to access and education. Work aimed at improving hand washing in resource constrained settings has shown that formative research with setting appropriate interventions are crucial in facilitating behavior change.[35] Given these findings prevention programs in this setting would be most efficacious if they addressed both access to, and understanding of soap use in relation to the socio-cultural context of the population.

Open defecation was more common than global estimates from resource-limited settings.[11] This high prevalence and the demonstrated risk reduction with access to improved sanitation facilities highlight the importance of this prevention point in programs addressing diarrheal disease in urban Kabul. Further, previous observational research from Kabul found an association between averted child deaths and latrine improvement programs which is in line with the findings here.[15,34] Setting specific evidence for public health interventions is vital in successful programs, and the results surrounding hand washing with soap and use of improved sanitation facilities should be used to focus diarrheal prevention strategies in Kabul.[36,37]

Rotavirus is postulated to be a major cause of diarrheal illness in Afghanistan.[38,39] In low-income settings, geographically similar to the study setting, rotavirus associated diarrhea predominates during colder seasons.[40] In this cohort the incidence of diarrheal illness was lowest during the winter period. This seasonal observation suggests a non-rotavirus diarrhoegenic predominance, however laboratory samples to identify microbiologic causes of enteric illnesses were not collected and the specific microbial profile in this setting could not be identified. Further microbiological data are required to investigate this finding

A trend of reduced risk of diarrheal illness was found among children from households that accessed water from an open well versus a piped source. This may relate to factors of water quantity and quality. Although improved water sources in the form of piped access have been shown to protect against diarrheal disease,[41] and approximately half of households in this study reported home piped access, no association with disease prevention was found with piped water. In Kabul water delivery is restricted and interruption of municipal water services is common.[42,43] This reduced delivery quantity perpetuates barriers to access and negates the intended benefits of improved water sources, and may explain the findings in this cohort. Alternatively, poor water quality due to contamination of municipal water could account for the observed findings. Therefore further studies to explore these hypotheses and disentangle the impact of water quantity and quality in this setting are needed.

## Limitations

Continuous surveillance for diarrheal illness was not done and subsequently, child episodes may have been missed. In this case, the calculated incidence rates would be an underestimation of the true burden of disease and non-differentially bias towards the null. Therefore any risk estimates observed would be an underestimation and thereby strengthening the associations found in this population.[44,45] Methodologically shorter recall periods have been shown to improve the accuracy of report of childhood diarrheal morbidity.[46,47] In the present study recall of stool frequency was limited to one day prior to assessment which should have served to increase the accuracy of identifying episodes of diarrheal disease.[48] Additionally the majority of diagnosed episodes in this study came from the clinic setting suggesting that caregivers were apt to address child illnesses, thereby making the probability of missed episodes less likely. This study gathered data on risk factors through participant reports that have the potential to suffer from recall bias. However, as the results are externally valid reproducing findings from other research settings, such bias is less likely.[33,34,49] Although seasonal variations in diarrhoegenic organisms are described and temporal trends in risks were observed, microbiologic samples were not collected and this limitation precludes etiologic seasonal analysis.[50,51] Subsequent studies should address this through concurrent microbiological sampling to better inform prevention initiatives. Lastly, confounding in relation to breastfeeding cannot be ruled out in this analysis. Although breastfeeding data at recruitment was available and child age as a proxy indicator was used, the study did not provide the ability to assess for the time varying effect of breastfeeding practices that may have altered disease risks throughout follow-up and this is a limitation of the analysis.[52]

## Conclusion

This study provides contemporary diarrheal incidence rates and evidence-based findings pertinent to prevention of childhood diarrheal illness in Afghanistan. Enhanced education and economic status were beneficial in this population and these findings are in line with multi-sector public health interventions to combat child illness. Maternal hand washing with soap and household use of improved sanitation facilities were found to be protective against diarrheal illness. Taking into account the low prevalence of hand washing with soap and high prevalence of open toilet use, these factors represent important prevention points for public health endeavors in Kabul that should be prospectively evaluated.

## Supporting Information

S1 Table Database key for S1 File. (DOCX)

S1 File (DTA)
