# Supplementary material for: Risk Factors Associated with Recurrent Diarrheal Illnesses among Children in Kabul, Afghanistan: A Prospective Cohort Study
Source: PLoS One. 2015 Feb 13;10(2):e0116342. doi: 10.1371/journal.pone.0116342 (PMC4332656; doi:10.1371/journal.pone.0116342)
Supplement: S1 Table — (DOCX) [file pone.0116342.s001.docx]

| **Database variable label** | **Database variable description** |
| --- | --- |
| id | unique participant identification number |
| date | date of evaluation |
| Source | assessment location |
| enroll | date of enrollment |
| dob | date of birth |
| age_vis | age at time of evaluation |
| days_risk | elapse time since prior evaluation |
| date_di | date of diarrheal illness |
| days_from_prev_di | elapse time since prior diarrheal illness |
| RF_Tx_drink_wat_how | type of drinking water treatment |
| di_repeat2 | indicator of diarrheal illness |
| date_last_seen | date of last evaluation during follow up |
| l2f | indicator for loss to follow up |
| mat_edu_bi | indicator of maternal education level |
| bed_share | indicator of child bed sharing |
| mom_h_wash_be_eat_soap | indicator of maternal hand washing with soap prior to eating |
| mom_drink_wat_sourc | indicator of type of water source utilized by the family |
| food_store | indicator of type of food storage utilized by the family |
| wat_source_dist | indicator of type of family water source |
| age_at | child age at time of evaluation |
| sz_cat | Indicator of season at time of illness |
| bf_curr_again | indicator of child feeding modality |
| mat_age_bi_rev | indicator of maternal age |
| sex_new | indicator of child gender |
| meas_indi | indicator of child vaccination for measles |
| poverty | indicator of family pverty status |
| timein_new_2 | entry time for diarrheal illness event |
| waz_full_indi_bi | indicator of malnutrition |
| toilet_cat_bi | indicator of type of toilet utilized by family |
